# Chest Imaging of Patients with Sarcoidosis and SARS-CoV-2 Infection. Current Evidence and Clinical Perspectives

**DOI:** 10.3390/diagnostics11020183

**Published:** 2021-01-27

**Authors:** Claudio Tana, Cesare Mantini, Francesco Cipollone, Maria Adele Giamberardino

**Affiliations:** 1 COVID-19 Medicine Unit and Geriatrics Clinic, “SS Annunziata” Hospital of Chieti, 66100 Chieti, Italy; 2 Department of Neuroscience, Imaging and Clinical Sciences, Institute of Radiology, “SS. Annunziata” Hospital, “G. D’Annunzio” University, 66100 Chieti, Italy; cesare.mantini@gmail.com; 3 COVID-19 Medicine Unit and Medical Clinic, “SS Annunziata” Hospital of Chieti, Department of Medicine and Science of Aging, “G D’Annunzio” University of Chieti, 66100 Chieti, Italy; fcipollone@unich.it; 4 COVID-19 Medicine Unit and Geriatrics Clinic, “SS Annunziata” Hospital of Chieti, Department of Medicine and Science of Aging and CAST, “G D’Annunzio” University of Chieti, 66100 Chieti, Italy; mag@unich.it

**Keywords:** sarcoidosis, SARS-CoV-2 infection, COVID-19, computed tomography, ultrasound, lung, pneumonia

## Abstract

The recent COVID-19 pandemic has dramatically changed the world in the last months, leading to a serious global emergency related to a novel coronavirus infection that affects both sexes of all ages ubiquitously. Advanced age, cardiovascular comorbidity, and viral load have been hypothesized as some of the risk factors for severity, but their role in patients affected with other diseases, in particular immune disorders, such as sarcoidosis, and the specific interaction between these two diseases remains unclear. The two conditions might share similar imaging findings but have distinctive features that are here described. The recent development of complex imaging softwares, called deep learning techniques, opens new scenarios for the diagnosis and management.

## 1. Introduction

The recent COVID-19 pandemic has dramatically changed the world in the last months, leading to a serious global emergency related to a novel coronavirus infection, characterized by high infectivity and severe clinical pictures affecting mainly the respiratory system [1].

Symptoms can affect both sexes and all ages ubiquitously, but it is actually unknown how in some cases there is a significantly higher degree of severity, with the presence of extensive lung involvement, respiratory failure, and need of invasive ventilation. Advanced age, blood group predisposition, and viral load have been hypothesized as some of the risk factors. Moreover, comorbidity, such as cardiovascular disease (CVD) seems to play a role, but these data do not explain the severity observed sometimes in younger and otherwise healthy age groups [1,2].

The role in patients affected with many other diseases, in particular immune disorders, also remains unclear, and although the COVID-19 infection seems to interact with the immune system by leading to an energetic T-cell activation and B-cell antibody production, it remains unknown how the interaction differs in patients with an altered immune system, in particular in cases characterized by impairment of the T-cell immunity and granuloma formation, as in sarcoidosis [3].

Interestingly, patients with sarcoidosis are characterized by lung predilection, and in some cases, the imaging features maybe similar to those of the patients with COVID-19 pneumonia. Sarcoidosis is a granulomatous disorder characterized by diffusion of noncaseating and non-necrotizing granulomas, lungs are the most affected site even though no organ is spared [4,5]. The role of infection from the novel coronavirus of patients having sarcoidosis is still largely unknown [3], and some authors suggest high attention for these patients, that are at risk of serious complications and clinical deterioration.

This narrative review aims at focusing on the current knowledge about imaging in sarcoidosis and COVID-19 infection. For this purpose, a literature search was performed on public databases (PubMed, Scopus). Search queries were the following: “Sarcoidosis” AND COVID-19 OR imaging OR computed tomography; “sarcoidosis” AND SARS-CoV-2 OR imaging OR computed tomography; “sarcoid lesions” AND COVID-19 OR imaging OR computed tomography; “sarcoid lesions” AND SARS-CoV-2 OR imaging OR computed tomography; “pulmonary” AND sarcoidosis OR sarcoid lesions OR COVID-19; “pulmonary” AND sarcoidosis OR sarcoid lesions OR SARS-CoV-2; “lung” AND sarcoidosis OR sarcoid lesions OR COVID-19; “lung” AND sarcoidosis OR sarcoid lesions OR SARS-CoV-2. 

Duplicate papers were considered only once. Papers with the main text not in English language and papers where the topic was not adherent to the theme of pulmonary sarcoidosis, COVID-19, and computed tomography were all excluded. 

## 2. Imaging Findings of Lung Involvement from Sarcoidosis

### 2.1. Imaging Findings of Sarcoidosis. The Scadding Classification at the Chest X-ray

**Figure 1:**
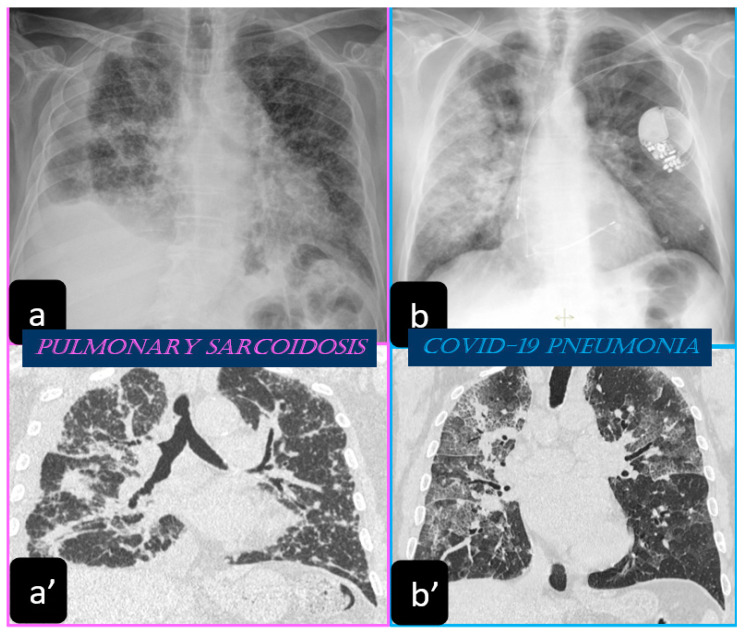
Chest X-ray of patients with pulmonary sarcoidosis shows the typical symmetric reticulonodular opacities (stage III). On the left side, the chest X-ray (**a**) and coronal (**a’**) high resolution computed tomography (HRCT) scans show the typical symmetric reticulonodular opacities of sarcoidosis (stage III). On the right side, the chest X-ray (**b**) shows the bilateral consolidation and ground glass haze. The coronal (**b’**) HRCT scan shows the presence of consolidation and ground-glass opacity with a superimposed interlobular septal thickening (“crazy-paving” pattern).

Traditionally, the chest X-ray has been used for several years to reveal the lung involvement of sarcoidosis. Four classes of involvement, according to the disease extension and severity, can be classified. Stage I defines the presence of bilateral hilar adenopathy, stage II shows the presence of bilateral hilar adenopathy with pulmonary infiltrates, while stage III shows the evidence of pulmonary infiltrates without the presence of overt hilar adenopathy (Figure 1a,a’), and stage IV shows the extensive lung involvement from pulmonary fibrosis. The chest X-ray has the limit of reduced resolution, and minimal parenchymal alterations can be missed at a routine lung examination [6].

### 2.2. Typical and Atypical Manifestation of Pulmonary Sarcoidosis at HRCT

**Figure 2:**
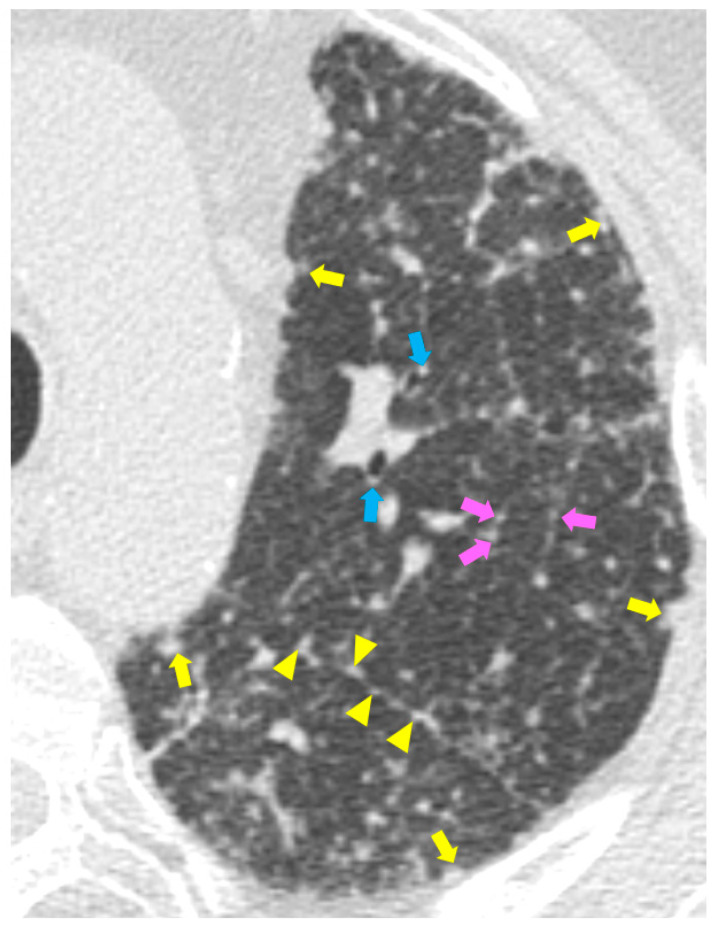
Computed tomography (CT) typical findings of sarcoidosis. The axial HRCT scan of the left lung in a patient with pulmonary sarcoidosis shows the typical perilymphatic distribution of micronodules along the peribronchovascular (blue arrows), subpleural interstitial space (yellow arrows), and interlobular septa (pink arrows). The yellow arrowheads show the typical subpleural distribution of the micronodules along the fissure.

**Figure 3:**
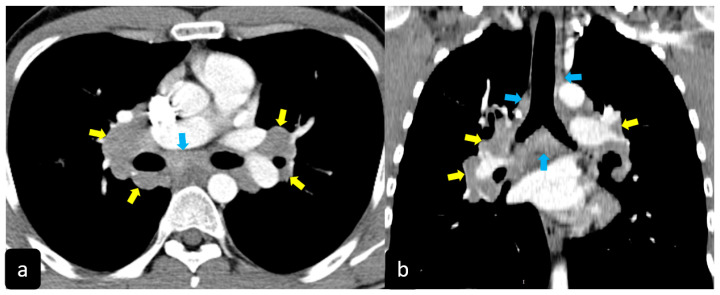
Axial (**a**) and coronal (**b**) CT scans in a patient with pulmonary sarcoidosis show the typical characteristic hilar ((yellow arrows) and mediastinal (blue arrows) bilateral lymphadenopathy.

**Figure 4:**
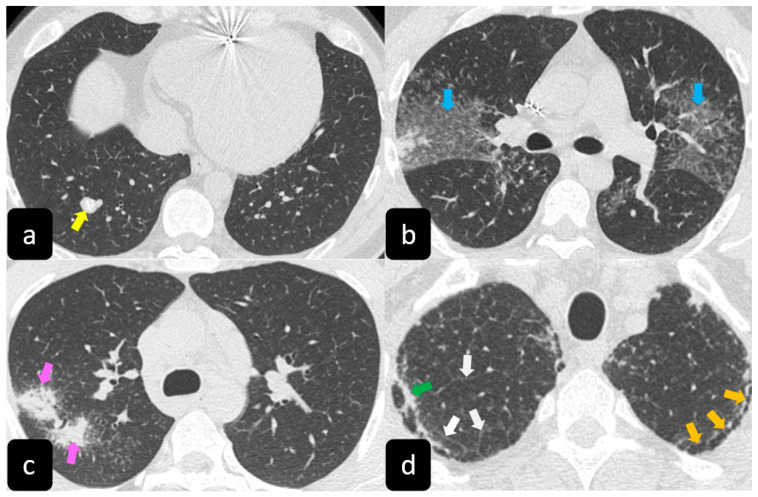
Axial HRCT scans show a solitary nodule (yellow arrow in (**a**)), ground-glass alveolar opacities (blue arrows in (**b**)), conglomerate masses “galaxy sign”(pink arrows in (**c**)), linear opacities (green arrow in (**d**)), thickened interlobar septa (white arrows in (**d**)), and honeycomb-like subpleural cysts (orange arrows in (**d**)).

High resolution computed tomography (HRCT) has a higher sensitivity than the chest X-ray and lymph node enlargement, commonly with a size of 2–5 mm, and the specific impairment of lungs are visualized more in detail. Parenchymal involvement can be minimal or characterized by bilateral parenchymal infiltrates that tend to merge into large and irregular pulmonary opacities. The most common and important parenchymal finding is the presence of micronodules (granulomas; 2–4 mm in diameter; well defined and bilateral) with a typical perilymphatic distribution along the peribronchovascular and subpleural interstitial space and interlobular septa (Figure 1a’ and Figure 2). CT is more sensitive than the chest x-ray in the identification of characteristic hilar (Figure 3a) and mediastinal (Figure 3b) lymphadenopathy [3]. A wide variety of less specific alterations can be found, such as unilateral or isolated lymphadenopathy, solitary nodules, confluent alveolar opacities, (the so-called alveolar sarcoid pattern), linear opacities, conglomerate masses, thickened interlobar septa, cysts, blebs, isolated bullae, or diffuse emphysema (Figure 4). Another typical imaging finding of sarcoidosis is the galaxy sign, a mass-like lesion, composed of numerous smaller coalescing granulomatous nodules, more concentrated in the center of the lesion (Figure 4c). The appearance of a central core with peripheral nodules is reminiscent of a globular cluster or galaxy [7,8].

**Figure 5:**
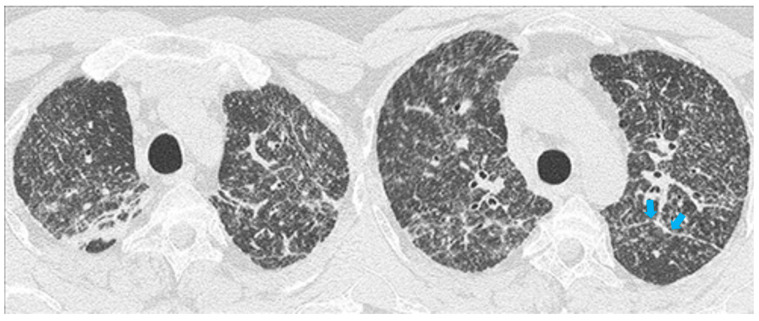
Axial HRCT scans show an advanced-stage sarcoidosis characterized by lung architectural distortion and traction bronchiectasis. Blue arrows show a left fissure displacement.

The presence of tracheobronchial abnormalities, atelectasis, or pleural involvement with thickening, effusions, calcification, pneumothorax, or plaque-like opacities such as aspergilloma and mycetoma can also be found [7,9,10]. Another atypical finding is represented by the ubiquitous presence of miliary opacities, that can mimic the parenchymal alterations observed in advanced tuberculosis [11,12]. Imaging findings can also be dominated, in an advanced stage, by architectural distortion, honeycombing-like opacities, traction bronchiectasis, and extended fibrosis (Figure 5). This group of findings represents a marker of poor outcome, being the evidence of irreversible alterations that are less likely to improve after therapy [13,14]. Clinical risk factors for fibrosis are illness severity, advanced age, history of smoking and alcoholism, prolonged ICU stay, and mechanical ventilation [15].

## 3. Imaging Findings of COVID-19 Pneumonia

### 3.1. Chest X-ray Findings

Chest radiographs show a limited value in the diagnosis of early stages mostly in the mild disease course. Rather, the HRCT findings may be present early even before the symptoms onset. Chest radiographs are useful in the intermediate to advanced stages of COVID-19 and for monitoring the rapid progression of lung abnormalities pneumonia, especially in critical patients admitted to intensive care units [16].

The most common chest X-ray features detected in COVID-19 cases were bilateral consolidation/ground glass haze (Figure 1b) and reticular interstitial thickening [16].

### 3.2. HRCT Findings of Lung Involvement from COVID-19

CT is now considered the main investigator for COVID-19, as it offers more sensitive results than the chest X-ray, especially in the initial assessment of the patients.

Lung involvement from COVID-19 is characterized by a broad spectrum of parenchymal alterations. 

**Figure 6:**
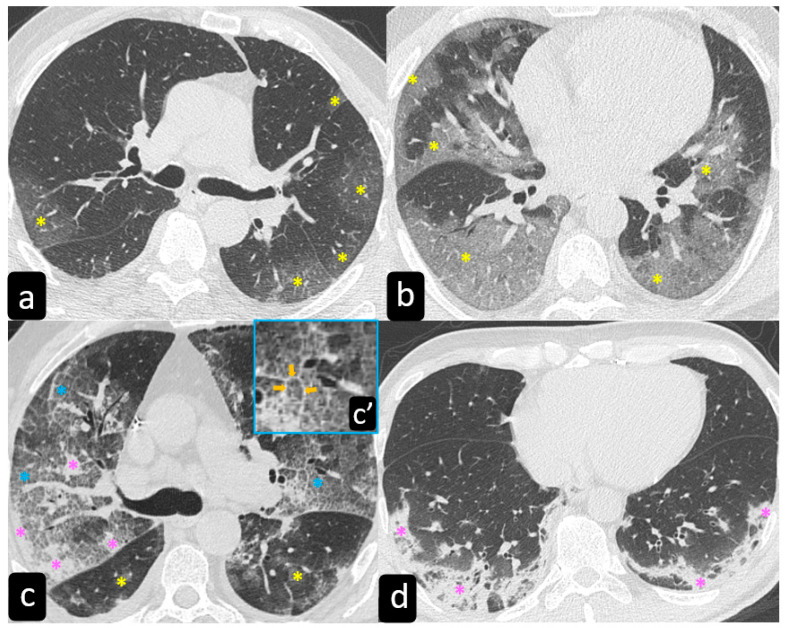
HRCT findings of COVID-19 pneumonia. Axial HRCT scans show bilateral ground glass opacities (yellow * in (**a**–**c**)) with or without posterior and peripheral lung consolidation (pink * in (**c**,**d**)) and “crazy-paving” pattern (blue * in (**c**)). “Crazy-paving” refers to the presence of ground-glass opacity with superimposed interlobular septal thickening (orange arrows in (**c’**)).

**Figure 7:**
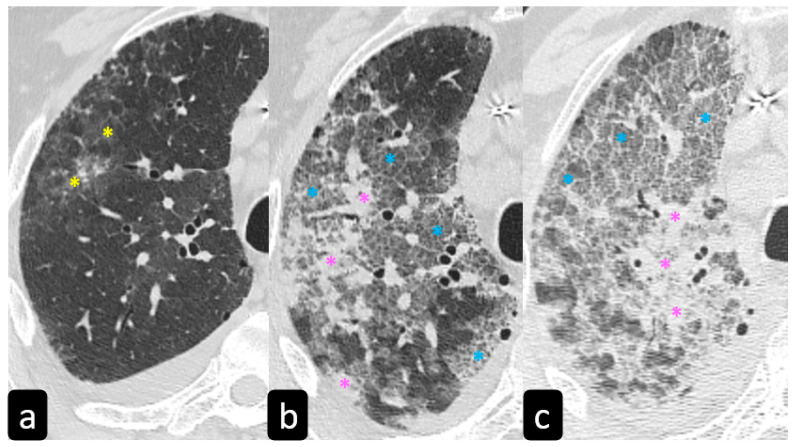
Patient presenting with fever and dyspnea. (**a**) At the first chest CT scan, day 2 showed patchy ground-glass opacities (yellow *). (**b**) At day 8, the lesions evolved intoconsolidations (pink *) and “crazy-paving” pattern lesions (blue *) and at peak time, day 14, became larger (**c**).

Typical findings that are hallmarks of the disease are represented by ground glass opacities (GGO) having a bilateral distribution, with or without posterior or peripheral lung consolidation (Figure 6) [17]. Usually, consolidations are an expression of disease progression after 1-3 weeks from the onset (Figure 7) [18].

**Figure 8:**
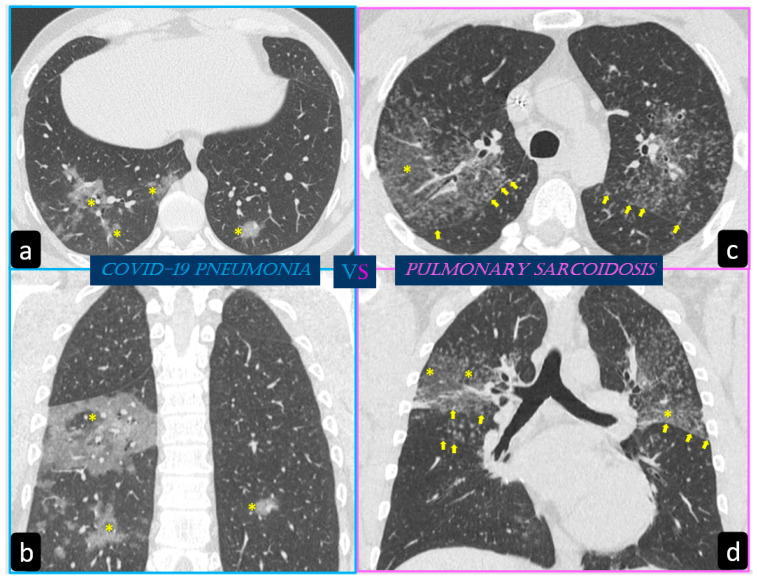
The images show a case of COVID-19 pneumonia (**a**,**b**) and a case of pulmonary sarcoidosis (**c**,**d**) both characterized by bilateral “ground glass opacities”. On the left side, axial (**a**) and coronal (**b**) HRCT scans show bilateral multiple ground glass opacities (yellow *) with peripheral distribution and lower lobe predilection (typical findings of COVID-19 pneumonia). On the right side, axial (**c**) and coronal (**d**) HRCT scans show (in addition to ground glass (GG) opacities) the typical perilymphatic distribution of micronodules also along the pulmonary fissures (yellow arrows) with upper lobes predilection (typical findings of pulmonary sarcoidosis).

By contrast, predominant GGO are rare and nonspecific findings in pulmonary sarcoidosis, being most typical in other diseases such as infections, pulmonary alveolar proteinosis, nonspecific interstitial pneumonia, alveolar hemorrhage, and also lung carcinoma [19]. In sarcoidosis, opacification is variably reversible, predicting the presence of alveolitis (Figure 8) [20,21]. As mentioned before, however, alveolar sarcoidosis is an atypical CT finding in sarcoidosis and without a specific pattern. In this setting, a hallmark of lung sarcoidosis can be the upper lobes preference, differently from COVID-19 pneumonia, which is characterized by a predominantly peripheral distribution and a lower lobes preference (Figure 8).

The second most observed CT finding in COVID-19 patients is the reticular pattern, characterized by interstitium thickening, mostly of intralobular lines and interlobular septa (Figure 6c,c’). Furthermore, HRCT shows the presence of little, linear opacities, which are more prevalent if the disease has a slow course [19,22]. Moreover, patients with sarcoidosis can show a predominant reticular pattern, characterized by a predominant interlobular septal thickening, that could be indistinguishable from that observed in COVID-19 [9].

By contrast, the association of ground glass opacities and thickening of interlobular lines and septa seem to be most specific of COVID-19, resembling the presence of irregular paving stones and translates into pictures characterized by the highest severity if associated with diffuse consolidations (Figure 7c) [17,18]. Sometimes, the “crazy-paving” pattern, mimicking paving stones at HRCT can be also a manifestation of alveolar sarcoidosis [23]. In patients with COVID-19 pneumonia, the initial CT findings are usually small subpleural patchy bilateral ground-glass opacities that subsequently grow larger and then the lesions develop to consolidation and a “crazy-paving” pattern. Subsequently, the lesions are reduced leaving subpleural parenchymal bands [18].

Nonspecific findings that can be observed both in COVID-19 and sarcoidosis, as well as in other lung diseases, are the presence of pleural and pericardial effusion, pleural thickening, air bronchogram, and the Halo sign defined as a ground glass opacification surrounding nodules or large masses [24,25]. While it has been hypothesized as a significance of reversibility for the Halo sign in active sarcoidosis [26], its role as a marker of progression or severity in COVID-19 is still uncertain [17].

Recently, lung ultrasound (LUS) has been largely employed in patients with COVID-19. With the progression of the disease, LUS can show findings such as consolidations, pleural line irregularities, and B-line artifacts at the bedside in the emergency room and in intensive care units. With portable US-devices, the patient can be evaluated in urgent conditions [27]. Ongoing studies are evaluating the diagnostic accuracy of LUS in COVID-19 patients [28], but preliminary data demonstrate that LUS can be effective to evaluate patients with SARS-COV-2 pneumonia, with the advantage of assessment of lesions mostly distributed by the dorsobasal terminal pleura, and accessible by LUS [29].

## 4. Hypothesis of Common Pathways of Pathogenesis and Mechanisms of Disease

The mechanism of the immune response occurring in patients with active sarcoidosis and infection from SARS-CoV-2 is largely unknown, but it has been hypothesized that it could involve common cellular pathways such as those that have a crucial role in regulating the mechanism of authophagy. Host-pathogen interactions at different points of the viral life cycle seem to be important for explaining in part the heterogeneity of clinical pictures that characterize COVID-19 [30]. In sarcoidosis, the presence of a sustained stimulation of the host immune system from the antigen exposition (infectious and non-infectious) has been reported as one of the main mechanisms of formation and maintenance of sarcoid granulomas [31,32].

Some authors have hypothesized that some constitutional defects of the regulation of macroautophagy in patients with sarcoidosis could predispose to more severe clinical pictures if they are infected from the novel SARS-CoV-2. Some viruses such as herpes and coronaviruses could indeed benefit from the dysregulation observed in sarcoidosis and bypass some autophagy steps [30]. Furthermore, the high affinity for the angiotensin-converting enzyme (ACE)2 protein, whose polymorphisms have been associated with different disease progression and severity in sarcoidosis, and the characteristic presence of typical lymphocytes reduction in both disorders, might be some of the mechanisms that could predispose to a high severity of the disease [30,33,34].

It has been long debated if the use of ACE inhibitors in COVID-19 patients or at risk of infection increases the risk of poor outcome, given the rationale that SARS-CoV-2 uses the ACE2 receptor to entry into target cells [35]. Recently, an increased risk of death associated with the use of ACE inhibitors and ARBs in COVID-19 patients was not found [36], but the risk in patients having sarcoidosis too, due to the co-existing polymorphisms affecting the ACE2 system might be increased [30].

The theory of a genetic predisposition to certain stages of severity in patients with sarcoidosis is interesting [37], but this hypothesis should be robustly investigated since there are no studies so far that have evaluated genetic patterns in patients having both active sarcoidosis and SARS-CoV-2 infection [30].

Therefore, it remains to be investigated which patients having sarcoidosis could be more predisposed not only to the lung deterioration from COVID-19 but also to the hypervascular response and hypercoagulability, typical of the severe form of the disease. Although the actual evidence on the use of immunosuppressive drugs such as chloroquine in COVID-19 is conflicting [38,39,40], and recent guidelines recommend against its use in COVID-19 patients, with the exception of clinical trials [41], some studies could be further aimed at investigating if patients with sarcoidosis, or in general those with autoimmune diseases, could benefit from immunosuppressive agents more than patients with only the SARS-CoV-2 infection [42,43]. Although the role of biological agents such as tocilizumab has been de-emphasized in moderately ill patients hospitalized for COVID-19, as this drug has not demonstrated efficacy to reduce the rate of intubation or death [43], the effectiveness in sarcoidosis patients has not been investigated. To date, there is no information on the potential immunosuppressive effects on active sarcoidosis [44]. Rather, there have been reports of isolated cases of patients treated with tocilizumab that paradoxically presented cutaneous sarcoidosis [45].

At present, the current mainstay of therapy for both sarcoidosis subjects and patients hospitalized for the SARS-CoV-2 infection remains the cortisone treatment. The positive effect of oral corticosteroids is well known, in improving symptoms, spirometry, and radiological findings in patients with acute flares of sarcoidosis [46]. Similarly, the use of dexamethasone has been associated with a 28-day mortality reduction among COVID-19 patients receiving either invasive mechanical ventilation or oxygen alone at randomization [47].

These data reflect and confirm the fact that the inflammatory response is a key step of pathogenesis in both conditions, regardless of the etiology, and therefore its interruption remains important to slow down the clinical deterioration [48]. It could be interesting to investigate any additional effect in patients having both conditions in the acute phase, not only one disease, since the interruption of the inflammatory cascade in both could result in a different clinical improvement and outcome.

## 5. Diagnostic Scenarios of Sarcoidosis Patients With SARS-CoV-2

The diagnosis of SARS-CoV-2 infection is achieved with the nasopharyngeal swab, which also reveals minimal traces of viral mRNAs. About 80% of the infected people are asymptomatic, and among patients with symptoms a variable quote manifests respiratory failure needing oxygenation or ventilation [49,50]. In these patients, chest HRCT is important to reveal the presence of parenchymal injury and to stratify the severity of lung impairment [51]. In patients with active sarcoidosis, it could be very hard to distinguish which damage is prevalent.

In this setting, four clinical scenarios could be revealed:

- Asymptomatic SARS-CoV-2 infection and stable sarcoidosis;
- Asymptomatic SARS-CoV-2 infection and active sarcoidosis;
- Symptomatic SARS-CoV-2 infection and stable sarcoidosis;
- Symptomatic SARS-CoV-2 infection and active sarcoidosis.

Laboratory exams are not helpful for the differentiation. The C-reactive protein (CRP) is usually elevated in both conditions and therefore, not specific to understand which form is predominantly active. Accordingly, laboratory exams can also reveal lymphopenia both in active sarcoidosis and infection from SARS-CoV-2. Another protein, ACE, is neither specific nor sensitive for the diagnosis of sarcoidosis, being elevated in several other non-sarcoid disorders [52,53], and its role as a biomarker for the diagnosis of COVID-19 has not been investigated so far [54,55].

### Role of HRCT in Discriminating Lung Involvement and the Diffusion of Deep Learning Techniques 

Chest HRCT can be useful to reveal some clues supporting a diagnosis of a prevalent active disorder. In particular, the presence of a “crazy-paving” pattern in a patient with the positive nasopharyngeal swab is diagnostic of lung impairment from COVID-19. Exceptionally, sarcoidosis can manifest with this feature, conversely the presence of small nodules distributed along vessels and the thickening of the bronchovascular bundles are virtually specific of sarcoidosis (Figure 2) [56].

The problem becomes complex if radiological findings are nonspecific or also commonly observed in other conditions such as ground glass opacities, reticular pattern, honeycombing, etc. [57]. However, a combination of multiple findings might orient towards a disorder rather than another one. 

In the last years, the progressive diffusion of artificial intelligence (AI) in medicine, particularly in radiology [58], the evolution of a processing software, and the introduction of deep learning techniques able to learn and recognize specific patterns such as humans, are increasing the capacity of detection of radiological findings with high accuracy, which are otherwise evident only with a high level of experience, reflecting a benefit in terms of the reduction of risk of errors and misdiagnosis, particularly if two or more conditions co-exist [56,59].

Therefore, novel algorithms could be designed to evaluate the probability of predominant impairment from sarcoidosis or COVID-19, according to predefined scores [60]. Such models could represent the key for distinction in the diagnostic approach to the conditions and a correct identification could be important to orient towards a correct therapy. 

Deep learning techniques are giving promising results in assessing the radiological features of COVID-19 [61,62,63,64]. In particular, a recent study found that a proposed algorithm reached a significantly higher overall diagnostic accuracy than that obtained by a simple radiologist observation both in COVID-19 pneumonia than in pneumonia from other causes [65].

Recently, an algorithm called CoroDet has been developed to distinguish two or more classes of lung involvement (COVID and normal; COVID, normal, and non-COVID pneumonia; COVID, normal, non-COVID viral pneumonia, and non-COVID bacterial pneumonia). 

The accuracy of classification was higher for all classes compared to the traditional method of analysis, in particular 99.1% for the two class, 94.2% for the three class, and 91.2% for the four class. The authors concluded that the CoroDet might be useful in clinical practice to predict the probability of SARS-CoV-2 infection, regardless of the results of the testing kit that could be unavailable in emergency conditions [61].

## 6. Conclusions

In a situation where the spread of COVID-19 is progressively affecting a large portion of the population, it remains necessary to investigate the effects and interactions of this disease in patients suffering from other pathologies. Since sarcoidosis is a disease with pulmonary predilection and has characteristics which are sometimes similar to those observed in COVID-19 patients, it is important to distinguish what aspects can be distinctive of one disease or the other. HRCT, and the related recent evolution of deep learning techniques, could provide additional key points for diagnosing which condition is prevalent, being important for the treatment and outcome of patients that can have both disorders in an acute phase of the disease.
